# miR199a-3p regulates P53 by targeting CABLES1 in mouse cardiac c-kit^+^ cells to promote proliferation and inhibit apoptosis through a negative feedback loop

**DOI:** 10.1186/s13287-017-0515-4

**Published:** 2017-06-05

**Authors:** Jingjin Liu, Yongshun Wang, Jinjin Cui, Meng Sun, Zhongyue Pu, Chao Wang, Wenjuan Du, Xinxin Liu, Jian Wu, Jingbo Hou, Shuo Zhang, Bo Yu

**Affiliations:** 1 Cardiology Department, Second Affiliated Hospital of Harbin Medical University, Harbin, Heilongjiang Province 150086 China; 2 Key Laboratories of the Education Ministry for Myocardial Ischemia Mechanisms and Treatment, Harbin, Heilongjiang Province China

## Abstract

**Background:**

MicroRNAs (miRNAs) have emerged as crucial factors that regulate proliferation and apoptosis of cardiac c-kit^+^ cells. Although much is known about their role in maintaining cardiac c-kit^+^ cell pluripotency, the mechanisms by which they affect cell fate decisions that are an essential part of the repair of heart failure remain poorly understood.

**Methods:**

Cardiac c-kit^+^ cells were obtained from Balb/c mice and cultured in vitro. Lentiviral vectors of miR199a-3p, its corresponding anti-miRNA, or short hairpin RNA against Cables1 were transfected into cells. The proliferation of cardiac c-kit^+^ cells was evaluated using EdU and flow cytometry. Furthermore, we examined cell apoptosis by flow cytometry under treatment with 200nM angiotensin II for 48 h. The levels of miR199a-3p and Cables1 mRNA were measured by quantitative real-time polymerase chain reaction (qRT-PCR). Western blot was performed to examine the expression of Cables1 and P53 proteins.

**Results:**

We demonstrated a significantly decreased expression of miR199a-3p in heart failure samples compared with healthy donors. Meanwhile, we identified miR199a-3p as a proliferation- and apoptosis-associated regulator impacted through Cdk5 and Abl enzyme substrate 1 (CABLES1) targeting, and also attributed their repression to P53 protein expression. We further demonstrated that P53 induced miR199a-3p expression and, in turn, miR199-3p decreased P53 activity.

**Conclusion:**

Collectively, our findings uncover one new mechanism by which P53 induced miR199a-3p expression and, in turn, miR199-3p decreased P53 activity. Therefore, miR199a-3p and P53 are coupled through CABLES1 and comprise a novel negative feedback loop that likely contributes to cardiac c-kit^+^ cell proliferation and apoptosis.

## Background

Heart failure, a frequent cause of death in the aging human population, is characterized by left ventricular remodeling and dilatation [1, 2] associated with activation of a fetal gene program triggering pathological changes in the myocardium associated with progressive dysfunction [3]. Several systems are involved in the induction of remodeling, including the well characterized increased activity of the renin–angiotensin–aldosterone system (RAAS) and sympathetic nervous system (SNS) [4]. MicroRNAs (miRNAs) are small noncoding RNAs that inhibit translation or promote mRNA degradation through binding to the 3′ untranslated region (UTR) of target mRNAs, resulting in “fine-tuning” of gene expression [5, 6].

Recently, several miRNAs have been implicated in heart failure [7, 8]. The miR199 family plays an important role in hypoxia-induced cell death through regulation of hypoxia-inducible factor-1a (HIF-1a) and the stabilization of the proapoptotic factor p53 [9]. Research has suggested that miR199 may have significant differential expression in the myocardium during heart failure. However, this research obtained different results, with some showing high expression [10, 11] and some significant underexpression [12–14]. The role of miR199a has been described in STAT-3 knockout mice which develop spontaneous heart failure [15]. Furthermore, the expression of miR590 or miR199a in the heart after infarction exerts a marked beneficial effect in reducing infarct size and in improving cardiac function [16]. Previous studies have shown that resident cardiac c-kit^+^ cells may be particularly suitable for restoring dead myocardium because these cells are endogenous components of the adult heart and they appear to be responsible for the physiological and pathological turnover of cardiac myocytes [17]. Furthermore, with c-kit dysfunction, myocardial angiogenesis and formation of heart tissue repair were limited.

Senescence and death of cardiac progenitor cells, which include cardiac c-kit^+^ cells, increased with age and contributed to the heart failure [18, 19]. Meanwhile, the upregulation of p53 may be critical in the modulation of heart failure [20, 21], and has also been shown to activate the miR199a-3p expression at the post-transcriptional level in induced pluripotent stem cells (iPSCs) [22].

Here, we hypothesized that the miR199a expression and activity in human failing myocardium may be a result of upregulation of P53 expression, and results in the survival of cardiac c-kit^+^ cells. This may ultimately offset P53 upregulation in heart failure.

## Methods

### Blood samples

Sixty patients with heart failure and 60 healthy adults from the Department of Cardiology, Second Affiliated Hospital of Harbin Medical University, were enrolled in our study between 2012 and 2014. Patients included in the present study had an ejection fraction cut-off of 45%. This study was approved by the Medical Ethics Committee of the Second Affiliated Hospital of Harbin Medical University, and written informed consent was obtained from all participants.

### Isolation of cardiac c-kit^+^ cells

The cardiac c-kit^+^ cells were isolated from the hearts of Balb/c mice (18–25 g) using a previously published method [23–25] with one minor modification. All of the Balb/c mice were obtained from the Laboratory Animal Science Department of the Second Affiliated Hospital of Harbin Medical University, Heilongjiang, People’s Republic of China. All experimental animal procedures were approved by the Local Ethics Committee for Animal Care and Use at Harbin Medical University in accordance with the guidelines of Directive 2010/63/EU of the European Parliament on the protection of animals used for scientific purposes and NIH guidelines. Briefly, the mice were injected with heparin (5000 IU/kg, intraperitoneally) 20 min prior to the initiation of the experimental protocol and were subsequently sacrificed through cervical dislocation. The heart was excised, and the aorta was rapidly cannulated. The cannulated heart was mounted on a Langendorff perfusion apparatus with constant flow, and the perfusion pressure was monitored. The heart was initially perfused with Ca^2+^-free Tyrode solution for 10 min to remove blood and was subsequently digested using 0.5 mg/ml collagenase (Sigma, St. Louis, MO, USA) and 0.5 mg/ml trypsin (Gibco, Invitrogen Inc., Carlsbad, CA, USA) at 37 °C for 30 min. The heart tissue was subsequently minced, and the cell suspension was filtered with a Steriflip (SCNY00100-1EA, Millipore Corp., Billerica, MA, USA). The cells were then incubated with FITC rat anti-mouse CD117/c-kit antibody (BD Biosciences, Franklin Lake, NJ, USA) and separated using MACS anti-FITC microbeads (Miltenyi Biotec, Bergisch Gladbach, Germany). Small round cells, containing most of the c-kit^+^ population, were obtained and cultured for 3–5 days in HyClone Dulbecco’s modified Eagle’s medium (DMEM)/F12 (Thermo Fisher Scientific, Waltham, MA, USA) containing fetal bovine serum (FBS), 10 ng/ml basic fibroblast growth factor (bFGF; PeproTech, Rocky Hill, NJ, USA), 10 ng/ml insulin-like growth factor (IGF; PeproTech), 10 ng/ml epidermal growth factor (EGF; PeproTech), and 10 ng/ml leukemia inhibitory factor (LIF; Sigma) at 37 °C. After recovery, the cells were used for experiments. We have previously shown that cardiac c-kit^+^ cells display some characteristics of the early cardiac phenotype. All animal experiments were approved by the Animal Ethics Committee (CEEA).

### Treatments

#### miRNA transfection

For miR199a-3p upregulation or knockdown, a miR199a-3p Lentivirus and anti-miR Lentivirus were purchased from GenePharma (Shanghai, China). Sequences were: mature miR199a-3p, 5′-ACAGUAGUCUGCACAUUGGUUA-3′; and anti miR199a-3p, 5′-AACCAAUGU-GCAGACUACUGUA-3′. We used virus titers ranging from 5 × 10^5^ to 1 × 10^7^ transducing units (TU)/ml.

#### CABLES1 gene and P53 gene transfection

In the present study, the recombinant lentivirus was produced by transient transfection of HEK293T cells using the calcium phosphate method; the virus was harvested at 48 and 72 h post-transfection and purified by centrifugation at 4 °C. The titer of the virus was 2 × 10^9^ TU/ml.

#### ShRNA transfection

The effective RNAi sequence targeting the CABLES1 gene was designed and screened at the Shanghai Bioladder Corporation (Shanghai, China). We selected sequences as target sequences for CABLES1: ShRNA-CABLES1: 5’-CACCGCTGCCATGCAAGAATATATGTTCAAGAGACATATATTCTTGCATGGCAGCTTTTTTG-3’; P53: shRNA-P53: 5’-TAATACGACTCACTATAGGGACGGCAGCGTGCAGCTCGACTCCAGTGGTAATCTACTTCAAGAGAGTAGATTACCACTGGAGTCTT-3’. The cDNA containing both the sense and antisense oligonucleotides of the targeting sequences was designed, synthesized, and cloned into the Plk0.1-GFP-SP6 vector to construct a lentiviral vector expressing ShRNA-CABLES1. We arrested the lenti-shRNA targeting packaging plasmids and co-transfected the plasmids into 293FT packaging cells. After 48 h of incubation, we collected the lentivirus released into the conditioned medium, filtered the medium, and used the supernatant to infect cardiac c-kit^+^ cells. After 2 weeks of antibiotic selection, stable clones were obtained and were subsequently confirmed through polymerase chain reaction (PCR) and DNA sequencing analysis.

#### Construction of luciferase reporter gene plasmids

Four types of oligonucleotides were used, referred to as the wild-type, mutation, oligonucleotides. The oligonucleotides were annealed with the following parameters: 95 °C for 5 min and 85 °C for 5 min, followed by 75 °C for 5 min and 70 °C for 5 min. The Psicheck-2 vector was cut by *Xho*I, and the fragment was isolated using the QIAquick Gel Extraction Kit (Qiagen, Venlo, Netherlands). The oligonucleotides and the fragments from the Psicheck-2 vector were ligated using T4 DNA ligase. Five clones from each type of oligonucleotide were cultured, and the plasmids were obtained. The successful constructs were confirmed through restriction enzyme digestion and sequencing.

#### Luciferase activity assay

Promoter activity was evaluated using a Dual Luciferase Reporter Assay Kit (Promega, Madison, WI, USA) according to the manufacturer’s instructions. The transfected cells were washed once with phosphate-buffered saline (PBS) and lysed in passive lysis buffer for 15 min with gentle agitation. LAR II (100 μl) was added to labeled luminometer tubes to complete the DLR™ assays. The luminometer was set to perform a 2-s premeasurement delay, followed by a 10-s measurement period for each reporter assay. A 20-μl sample of the cell lysate was carefully transferred to the luminometer tube containing LAR II, and the solution was mixed after pipetting 2–3 times. The tubes were placed in the luminometer and the intensity was measured. Subsequently, the sample tubes were removed from the luminometer, and 100 μl Stop & Glo® Reagent was added. After brief vortexing, the samples were again measured in the luminometer.

#### Flow cytometry

##### Apoptosis detection

The cardiac c-kit^+^ cell apoptosis was assessed using the annexin V-FITC apoptosis detection kit according to the manufacturer’s instructions (BD Biosciences). Briefly, the cardiac c-kit^+^ cells were grown in the presence of 200nM angiotensin II for 48 h. The cardiac c-kit^+^ cells were cultured in DMEM/F12 with 10% FBS as negative controls. Cells were harvested and washed once in PBS and resuspended in buffer and incubated with annexin V-FITC in the dark at room temperature for 30 min. Cells were then washed once with PBS and resuspended in buffer supplemented with propidium iodide (PI). A total of 10,000 events were acquired using a BD LSRII flow cytometer (BD Biosciences) and the data were analyzed using BD FACSDiva™ Software. Flow cytometry was performed in duplicate using cells from three independent experiments.

#### Cell proliferation assay

The proliferation of cardiac c-kit^+^ cells was determined through 5-ethynyl-2-deoxyuridine (EdU) incorporation (RiboBio, Guangzhou, China). The cells were fixed and stained after incubation according to the manufacturer’s instructions. The proliferation rate was calculated after normalizing the number of EdU-positive cells to the number of DAPI-stained cells in five random fields.

For cell cycle analysis, the cells were harvested at 48 h after transfection with the miR199a-3p or anti miR199a-3p, then washed twice with PBS and fixed in 75% ethanol overnight. The next day, the cells were washed twice with PBS and incubated in RNaseA (20 mg/ml) at 37 °C for 30 min, followed by staining with PI (0.5 mg/ml) at 4 °C for 30 min. The cells were subsequently washed and resuspended in 500 ml PBS, followed by the detection of the DNA contents using a Becton-Dickinson flow cytometer (BD Diagnostics, Sparks, MD, USA).

#### Quantitative real-time PCR

Total RNA was isolated with TRIzol (Invitrogen Inc., Carlsbad, CA, USA) from the cells subjected to the different experimental conditions according to the manufacturer’s instructions. After pretreatment using RNase-free DNase I, 2 μg total miRNA was subjected to the protocols of the miScript Reverse Transcription Kit and the miScript SYBR Green PCR kit (Qiagen, Venlo, the Netherlands), and the Transcriptor First Strand cDNA Synthesis Kit and FastStart Universal SYBR Green Master Mix (ROX) were used for reverse transcription. Gene amplification was confirmed after calculating the melting temperatures (Tm) for the products from the melting peak curve (2^dF/dT^ versus temperature).

All amplicons were collected and confirmed through agarose gel electrophoresis and sequencing. A cross-point versus logarithmic concentration standard curve was generated using serial dilutions of one of the cDNA samples or known concentrations of plasmid DNA with a gene insert. Negative controls were included using cDNAs synthesized in the same manner as described above, but without reverse transcriptase. Each cDNA sample was run in triplicate. The data were averaged and standard deviations were calculated. The GAPDH gene was used as a standard control. U6 was employed for miR199a-3p template normalization. The primers used in this study and PCR conditions are: CABLES1 primers (5′-CTCCGGAGATGTCGAGCTCTCTCAGGTTC-3′; 5′-GCTCCCTGGGTGCCGGCTGCCCGGCCTATGGAG-3′), P53 primers (5′-CAAGCTTATGCCCCCAGGGAGCACTAAGCGAGCA-3′; 5′-TCTCGAGTCAGTCTGAGTCAGGCCC-3′;) and β-actin primers (5′-GCTCGTCGTCGACAACGGCTC-3′; 5′-CAAACATGATCTGGGTCATCTTCTC-3′).

#### TOPFlash reporter assay

The wild-type TOPflash P53 LUC reporter (200 ng; Addgene, Inc., Cambridge, MA, USA) was co-transfected with the miR199a-3p and anti-miR Lentivirus, along with 5 ng of a Renilla LUC reporter plasmid using Oligofectamine (Invitrogen) in cardiac c-kit^+^ cells (60% confluency). Transfection was performed according to the manufacturer’s protocol. The transfected cells were incubated at 37 °C for 24 h and assayed for relative luciferase activity normalized to Renilla values.

#### Western blotting

Protein samples were denatured at 95 °C for 5 min before being loaded onto an SDS-polyacrylamide gel (reducing or nonreducing, 4–15%) and run under standard conditions. The proteins were then transferred to polyvinylidene difluoride membranes (PVDF; Millipore Corp., Billerica, MA, USA). The membranes were blocked with 5% nonfat milk at room temperature for 1 h in Tris-buffered saline containing Tween 20 (TBST). Primary antibodies against CABLES1 (abcam ab75537) and P-53 (Cell Signaling Technology), and α-actin (Santa Cruz sc-10731) were incubated overnight with the membranes at 4 °C. The membranes were subsequently incubated for 60 min with peroxidase-conjugated Affinipure goat anti-rabbit IgG (H + L) and anti-mouse IgG (H + L)-labeled secondary antibodies diluted 1:2000. The membranes were washed in TBST with 0.5% Tween-20 before ECL detection with the BeyoECL Plus (Beyotime Institute of Biotechnology, Haimen, China). After exposure of an X-ray film, the blot was stripped in 5 ml stripping buffer (CoWin Biotech, Beijing, China) for 15 min at room temperature and hybridized with an antibody against α-actin for normalization. Densitometric analysis of the resultant protein bands was performed using the Tanon Gel Imaging System (Shanghai Tanon Co. Ltd., Shanghai, China).

#### Statistical analysis

Statistical analysis was performed using SPSS 18.0 (SPSS Inc., Chicago, IL, USA). The measurements are presented as the means ± SD. Comparisons of all pairs were performed using Student’s *t* test or the least significant difference (LSD) test, as appropriate. *P* values of <0.05 were considered significant. The correlation between miR199a-3p expression and the protein levels of the target genes was examined through Pearson’s correlation analysis. Binary regression and receiver-operating characteristic (ROC) analyses were used to test the diagnostic utility of miRNAs. The results are shown as the mean ± SD of at least three separate experiments.

## Results

### Expression of miR199a is downregulated in heart failure patients

We performed RT-PCR in plasma samples from 60 heart failure patients and 60 healthy donors to analyze the expression of miR199a-3p and miR199a-5p. The baseline characteristics of study participants are shown in Table 1.

**Table 1:** Baseline characteristics of healthy volunteers and heart failure patients

	Healthy volunteers (*n* = 60)	Heart failure (*n* = 60)
Age (years)	68.43 ± 12.11	63.91 ± 11.75
Gender (male)	55%	58.3%
Ejection fraction (%)	54.7 ± 4.33	35.6 ± 9.4
Left ventricular internal dimension diastole (mm)	54.9 ± 7.75	59.5 ± 11.7
Left ventricular internal dimension systole (mm)	38.4 ± 6.5	49.8 ± 10.8
NT-proBNP (pmol/L)	137.08 (14–468)	6786.3 (576–24072)
hs-troponin T (pmol/L)	0.2901 (0.002–2.23)	1.48 (0.012–17.322)

Values are shown as arithmetic mean ± standard deviation, or geometric mean (range)

**Fig. 1:**
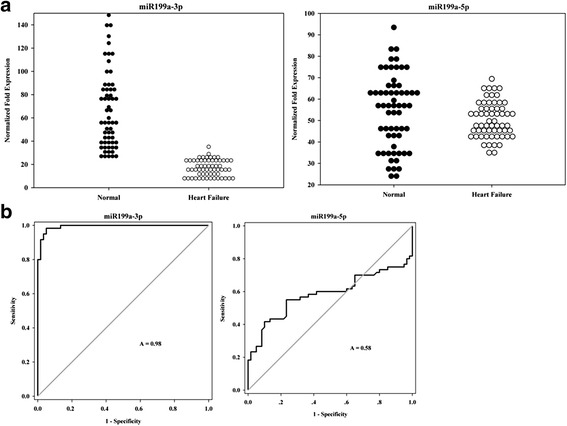
Significantly decreased miR199a-3p expression was detected in heart failure patients. **a** Expression of miR199a-3p and miR199a-5p in the plasma from 60 heart failure patients and 60 healthy donors was detected by RT-PCR. U6 was used as the expression control. Each real-time PCR assay was performed in triplicate. **b** ROC curve analysis of miR199a-3p and miR199a-5p expression in patients. Area under the curve (*A*) sensitivity and specificity were 0.98 and 0.58, respectively

We observed a significantly decreased expression of miR199a-3p in heart failure samples compared with healthy donors (Fig. 1a). However, no significant change in the expression of miR199a-5p was detected.

ROC curve analysis suggested that the expression levels of miR199a-3p could be used as a strong diagnostic predictor of heart failure, with an area under the curve (AUC) of 0.98 (Fig. 1b).

### miR199a-3p is involved in regulation of cardiac c-kit^+^ cell proliferation and apoptosis

**Fig. 2:**
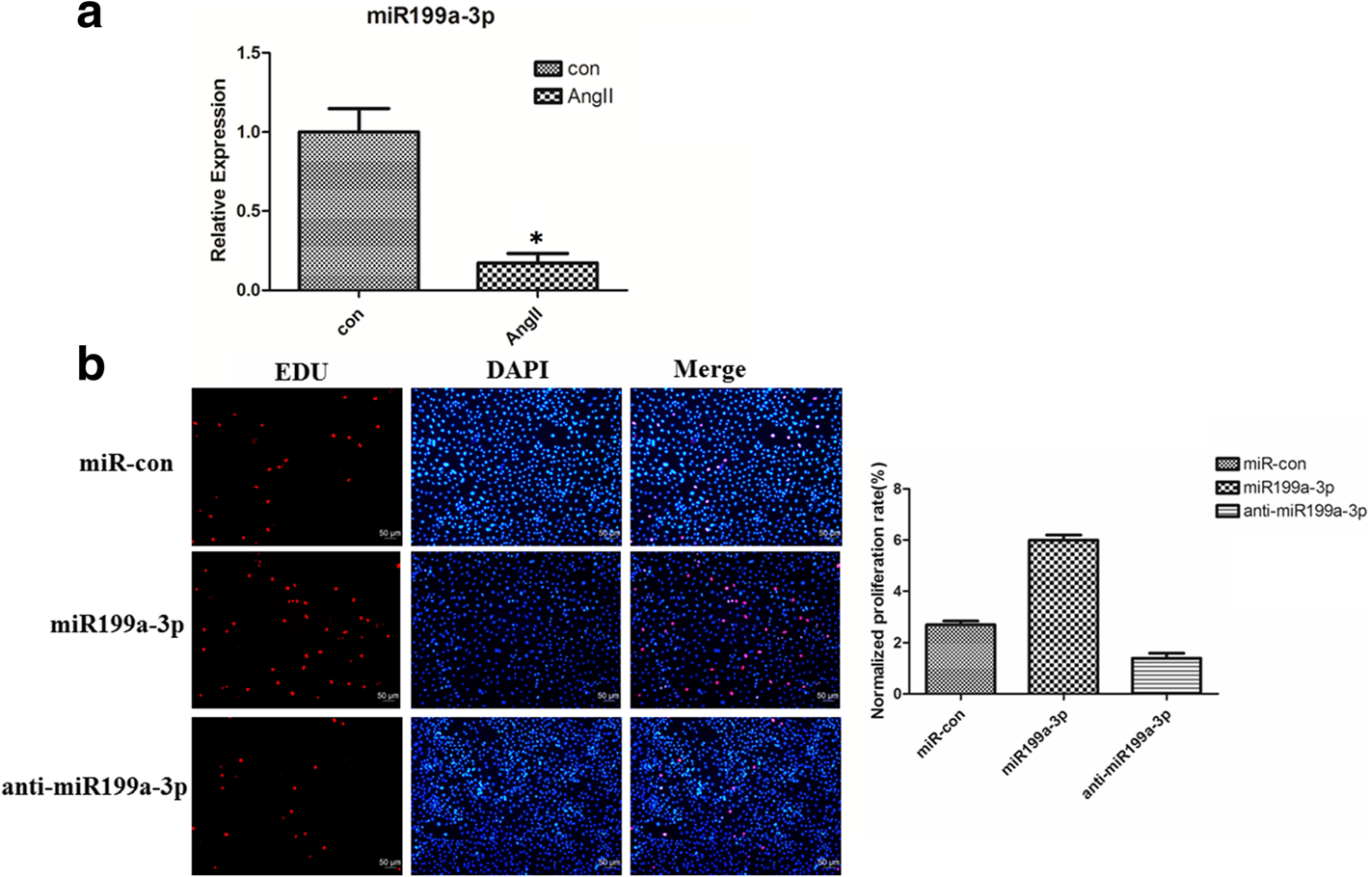
miR199a-3p is involved in the regulation of cardiac c-kit^+^ cell proliferation and apoptosis. **a** Analysis of total RNA to determine the relative expression of miR199a-3p through qRT-PCR in cardiac c-kit^+^ cells at 48 h after treatment with 200 nM angiotensin II (*AngII*). Expression was normalized to U6 (*p < 0.05). **b** The cells were stained with EdU and DAPI after transfection with the miR199a-3p and anti-miR199a-3p. *Con* control

**Fig. 3:**
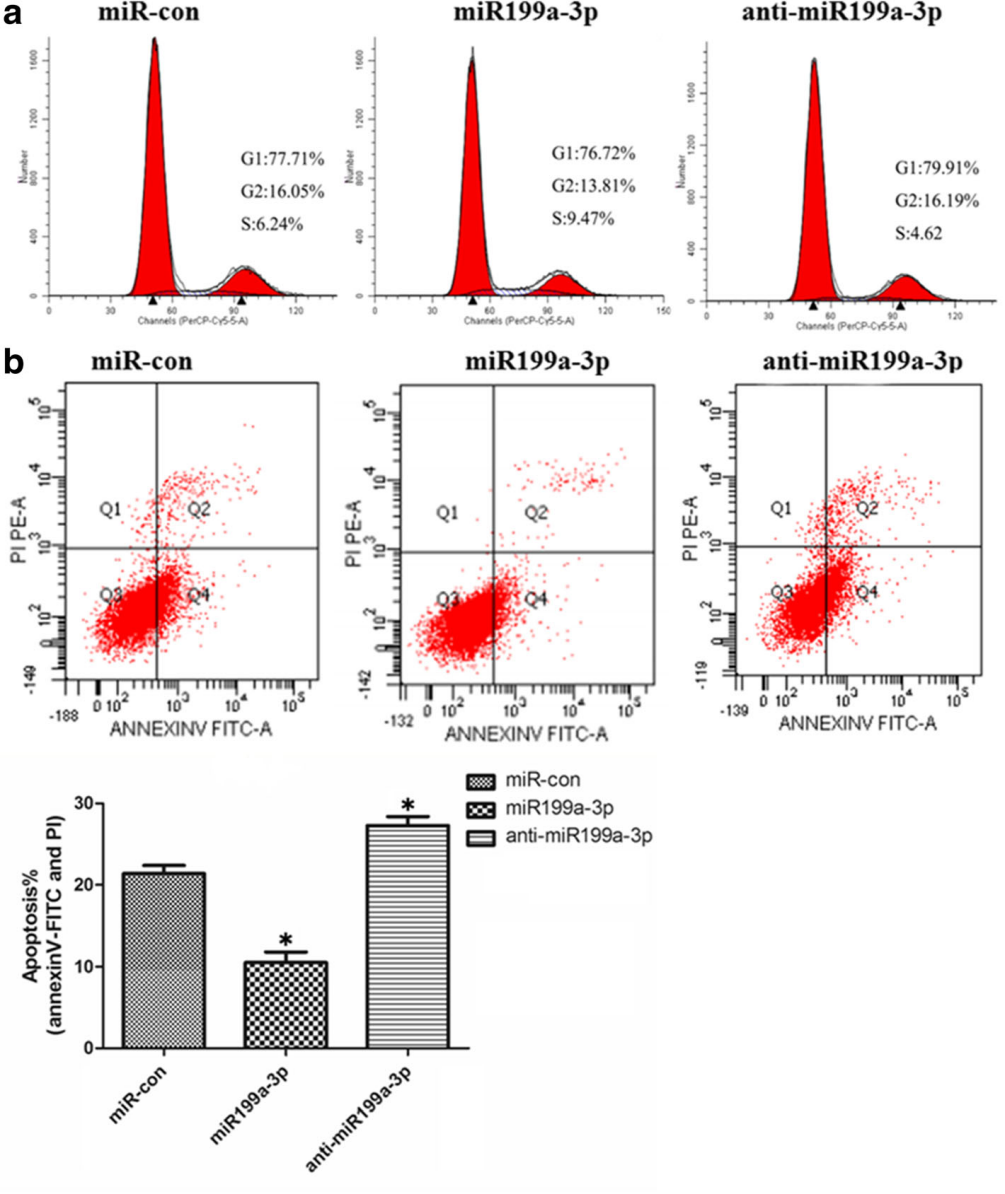
miR199a-3p is involved in the regulation of cardiac c-kit^+^ cell proliferation and apoptosis. **a** Cell cycle distribution of cardiac c-kit^+^ cells after transfection with the miR199a-3p and anti-miR199a-3p for 48 h. **b** Representative annexin V/PI flow cytometry analysis of cardiac c-kit^+^ cells. Quantification of analysis of annexin V^+^/PI^+^ cardiac c-kit^+^ cells by flow cytometry. The results are shown as the means ± SD of at least three separate experiments. *Con* control, *PI* propidium iodide (*p < 0.05, compared to miR-con)

To examine whether miR199a-3p affects cell proliferation and apoptosis in cardiac c-kit^+^ cells we first tested the miR199a-3p expression after 200 nM angiotensin II treatment for 48 h in cardiac c-kit^+^ cells. We found that miR199a-3p had lower expression in cardiac c-kit^+^ cells after apoptosis (Fig. 2a). The functional activity of miR199a-3p was assessed in cardiac c-kit^+^ cells after forced expression of this miRNA or its corresponding anti-miRNA using lentiviral vectors. After transfection, we evaluated the proliferation of cardiac c-kit^+^ cells using EdU and flow cytometry. The EdU results showed that overexpression of miR199a-3p was able to promote cell proliferation (6.1 ± 0.28%) compared with the control group (2.4 ± 0.25%), whereas the anti-miR199a-3p group (1.7% ± 0.33%) exhibited lower proliferation (Fig. 2b). We use flow cytometry to examine the DNA context in miRNA-treated cardiac c-kit^+^ cells. We observed that transfection of the miR199a-3p promoted cell proliferation to a greater extent (9.55 ± 0.66%, S phase) compared with the control group (6.15 ± 0.31%), whereas silencing miR199a-3p decreased cell proliferation (4.33 ± 0.58%, S phase) (Fig. 3a).

We treated cardiac c-kit^+^ cells with 200nM angiotensin II for 48 h and examined cell apoptosis by flow cytometry. The data showed that miR199a-3p protected cardiac c-kit^+^ cells from angiotensin II-induced apoptosis (11.2% ± 2.1%) compared with control group (22.7% ± 1.1%) (Fig. 3b).

Therefore, these data showed that miR199a-3p promotes cell proliferation and inhibits apoptosis in cardiac c-kit^+^ cells.

### CABLES1 is a target for miR199a-3p

To address the mechanism through which miR199a-3p regulates cardiac c-kit^+^ cell survival, we examined the predicted targets of miR199a-3p. The putative target sites of miR199a-3p were predicted using TargetScan, microRNA and PicTar software. Among all of the possible targets, several potential ones meeting this criterion were determined to be the top putative targets.

From these genes, we selected Cdk5 and Abl enzyme substrate 1 (CABLES1) for further study since this protein is an adaptor protein that links the cyclin-dependent kinase (Cdk) to the nonreceptor tyrosine kinases and regulates the activity of Cdks by enhancing their Y15 phosphorylation [26, 27].

**Fig. 4:**
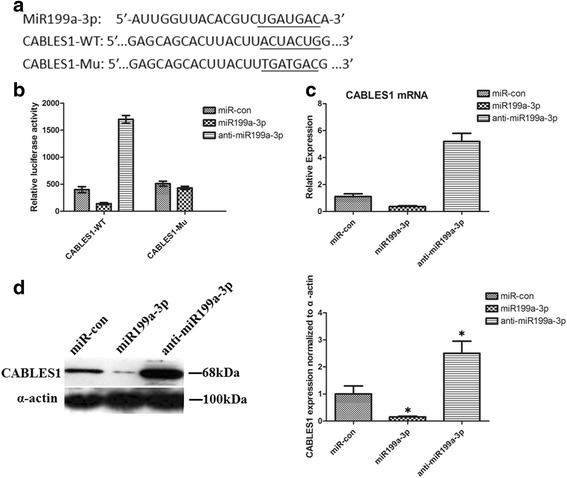
CABLES1 is a target of miR199a-3p in cardiac c-kit^+^ cells. **a** The nucleotide sequence of miR199a-3p, the predicted binding sites of miR199a-3p, and the mutated nucleotides (*underlined*) in the 3′ UTR of CABLES1. **b** Dual luciferase activity in transfected cardiac c-kit^+^ cells. **c** Regulation of endogenous expression of CABLES1 by treatment with the miR199a-3p lentiviral vectors and anti-miR199a-3p. Analysis of total RNA to determine the relative expression of miR199a-3p targets through qRT-PCR in cardiac c-kit^+^ cells at 48 h after transfection. Relative expression levels were normalized to GAPDH levels. **d** Western blot analysis of proteins from cardiac c-kit^+^ cells transfected with the miR199a-3p lentiviral vectors and anti-miR199a-3p. The protein profiles were normalized to α-actin. *CABLES1* Cdk5 and Abl enzyme substrate 1, *Con* control (*p < 0.05, compared to miR-con)

There are possible binding sites in the CABLES1 3’ UTR. The miR199a-3p binding site in the seed sequences within the 3’ UTR of CABLES1 mRNA is illustrated in Fig. 4a. To determine whether miR199a-3p directly regulates CABLES1, we performed luciferase reporter experiments and observed that the luciferase activity of CABLES1-WT was markedly reduced after transfection with the miR199a-3p lentiviral vectors for 24 h. However, single mutations completely abolished the repression induced by miR199a-3p (Fig. 4b), indicating that miR199a-3p could specifically target the binding sites in the 3’ UTR of CABLES1.

We examined mRNA expression for each target in response to miR199a-3p and anti-miR199a-3p. We observed that CABLES1 mRNA expression was significantly changed (Fig. 4c). We examined the protein expression levels of CABLES1 in response to miR199a-3p. We observed that CABLES1 protein levels were significantly decreased by treatment with the miR199a-3p lentiviral vectors and increased by treatment with the anti-miR199a-3p (Fig. 3d). Together, these results indicated that CABLES1 is a direct target of miR199a-3p in cardiac c-kit+ cells.

### miR199a-3p regulates cardiac c-kit^+^ cell proliferation and apoptosis through targeting CABLES1

**Fig. 5:**
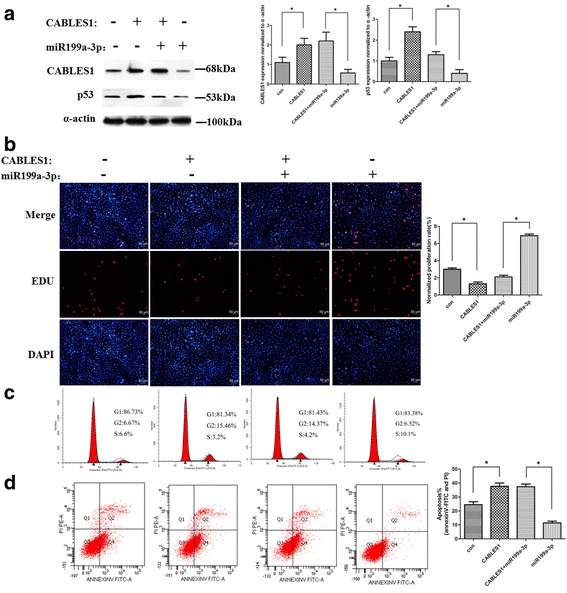
miR199a-3p regulates cell proliferation and apoptosis through targeting CABLES1. **a** Western blot analysis of proteins from cardiac c-kit^+^ cells transfected with the miR199a-3p and CABLES1 lentiviral vectors (*p < 0.05). The protein profiles were normalized to α-actin. **b** The cells were stained with EdU and DAPI (*p < 0.05). **c** Cell cycle distribution of cardiac c-kit^+^ cells after transfection with the miR199a-3p and CABLES1 lentiviral vectors. **d** Representative annexin V/PI flow cytometry analysis of cardiac c-kit^+^ cells and analysis of annexin V^+^/PI^+^ cardiac c-kit^+^ cells by flow cytometry. *CABLES1* Cdk5 and Abl enzyme substrate 1, *Con* control, *PI* propidium iodide (*p < 0.05)

To determine whether the regulation of proliferation and apoptosis of cardiac c-kit^+^ cells through miR199a-3p is directly mediated by CABLES1, we performed rescue experiments by transfecting the miR199a-3p and CABLES1 lentiviral vectors into cardiac c-kit^+^ cells and examining CABLES1 expression levels through Western blotting (Fig. 5a).

The EdU results show that the CABLES1 transfection mitigated the cell amplification (1.8 ± 0.31% and 2.1 ± 0.34%, respectively) which results from miR199a-3p treatment or negative control (2.6 ± 0.21% and 6.6 ± 0.28%, respectively), and results in reduced cell proliferation (Fig. 5b). Similar results were observed in the flow cytometry test. The results suggested that cardiac c-kit^+^ cells express lower proliferation (3.2 ± 0.4% and 4.2 ± 0.3%, respectively) in combination transfection of miR199a-3p and CABLES1 than the control group (6.5 ± 0.6% and 10.5 ± 0.7%, respectively) (Fig. 5c). Furthermore, the apoptosis by angiotensin II was much more increased in the combination group (37.7 ± 1.9%) or the CABLES1 transfection group (38.6 ± 2.1%) than the miR199a-3p group (10.8 ± 1.3%) or the negative control (25.7 ± 2.3%) (Fig. 5d).

**Fig. 6:**
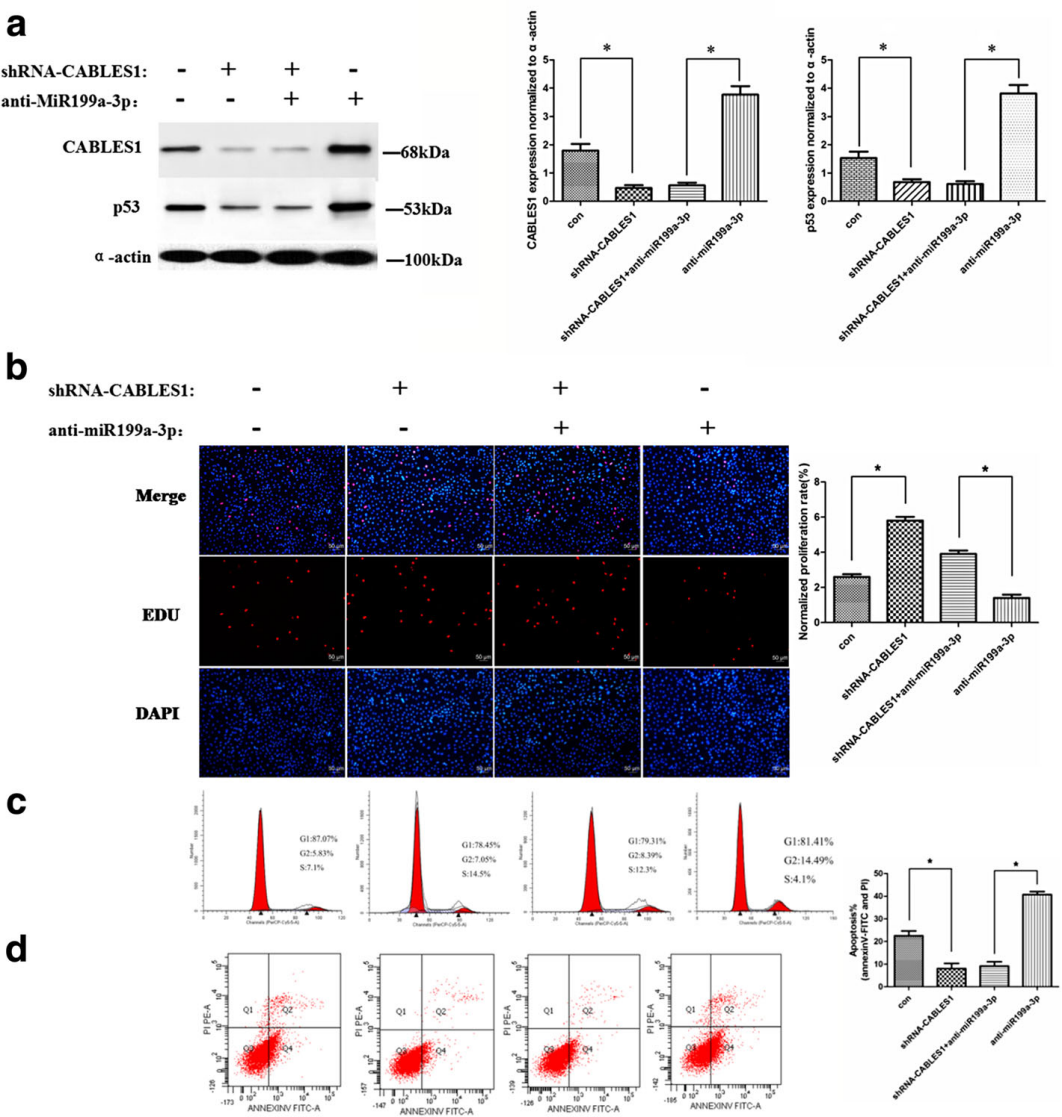
miR199a-3p regulates cell proliferation and apoptosis through targeting CABLES1. **a** Western blot analysis of proteins from cardiac c-kit^+^ cells transfected with the anti-miR199a-3p and shRNA-CABLES1 lentiviral vectors (*p < 0.05). **b** The cells were stained with EdU and DAPI (*p < 0.05). **c** Cell cycle distribution of cardiac c-kit^+^ cells after transfection with the anti-miR199a-3p and shRNA-CABLES1 lentiviral vectors. **d** Representative annexin V/PI flow cytometry analysis of cardiac c-kit^+^ cells and analysis of annexin V^+^/PI^+^ cardiac c-kit^+^ cells by flow cytometry (*p < 0.05). *CABLES1* Cdk5 and Abl enzyme substrate 1, *Con* control, *PI* propidium iodide

We performed rescue experiments by transfection with the combination of anti-miR199-3p and shRNA-CABLES1 in cardiac c-kit^+^ cells and detected the protein expression by Western blotting (Fig. 6a).

EdU and cell cycle assays demonstrated that the shRNA-CABLES1 group was more proliferative (5.8 ± 0.31% and 4.0 ± 0.28, respectively) than the inhibitor control group (2.2 ± 0.23% and 1.7 ± 0.21%, respectively) (Fig. 6b). The proliferation results also suggested that cells transfected with the combination of the antimiR199a-3p and shRNA-CABLES1 (14.5 ± 0.41% and 12.3 ± 0.27%, respectively) showed higher proliferation than those treated with the anti-miR199a-3p or negative control (7.1 ± 0.33% and 4.1 ± 0.18, respectively) (Fig. 6c), whereas apoptosis by angiotensin II was restricted in the combined transfection group (9.3 ± 1.8%) or shRNA-CABLES1 transfection group (8.2 ± 2,1%) compared with antimiR199a-3p treatment (39.8 ± 1.5%) or negative control (23.1 ± 1.9%) (Fig. 6d).

These data confirmed that the effects of miR199a-3p on proliferation and apoptosis in cardiac c-kit^+^ cells could be attributed to the target protein CABLES1.

### The regulation of miR199a-3p on cell proliferation and apoptosis is involved in P53

As CABLES1 connects Cdk2 and Wee1 which results in increased phosphorylation of Cdk2 at Y15, decreased kinase activity, and reduced cell proliferation. CABLES1 interacts with p53 and p73 resulting in the induction of cell death [28]. Furthermore, suppression of p53 expression in CABLES1 morphants suggests that the phenotype of CABLES1 morphants is due in part to p53-dependent apoptosis [29].

To identify the mechanisms associated with miR199a-3p and P53 in cardiac c-kit^+^ cells, we examined P53 expression in response to miR199a-3p and CABLES1.

**Fig. 7:**
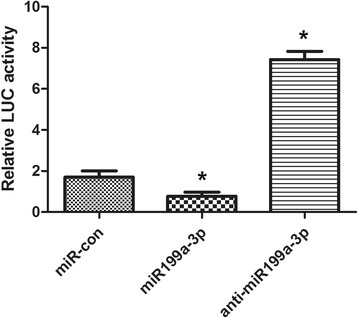
The regulation of miR199a-3p on cell proliferation and apoptosis is involved in P53 (*p < 0.05, compared to miR-con). The WT Wnt TOPflash reporter was transfected into cardiac c-kit^+^ cells together with the mimic-control (*con*), miR199a-3p, and anti-miR199a-3p. Relative luciferase (*LUC*) activity was measured and plotted

TOPflash reporter assay was performed to confirm that P53 was involved in the effects of miR199a-3p in cardiac c-kit^+^ cells. The results showed that P53 activity was decreased 48% in the miR199a-3p group and increased 4.2-fold in the anti-miR199a-3p group (Fig. 7).

Western blotting analysis revealed that treatment with miR199a-3p decreased endogenous P53 expression. The same result also appears in the CABLES1 transfection groups (Fig. 5a).

We performed experiments by transfection with the combination of anti-miR199-3p and shRNA-CABLES1 in cardiac c-kit^+^ cells and detected the P53 protein expression by Western blotting (Fig. 6a).

These data demonstrated that miR199a-3p could promote cell proliferation and inhibit cell apoptosis through regulating P53.

### A negative feedback loop comprising miR199a-3p, CABLES1, and P53 is involved in cardiac c-kit^+^ cells

**Fig. 8:**
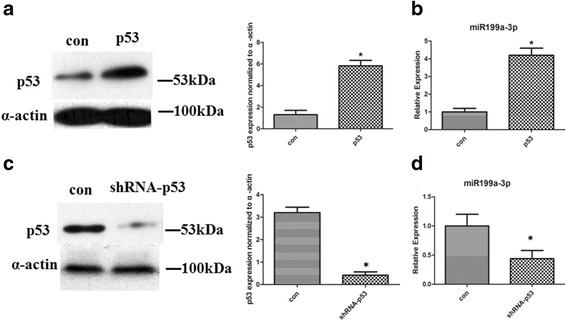
A negative feedback loop comprising miR199a-3p, CABLES1, and P53 is involved in cardiac c-kit^+^ cells. **a** Western blotting analysis of proteins from cardiac c-kit^+^ cells transfected with the P53 lentivirus. The protein profiles were normalized to α-actin. **b** Expression of miR199a-3p mRNA in cardiac c-kit^+^ cells transfected with the P53 lentivirus. **c** Western blotting analysis of P53 proteins from cardiac c-kit^+^ cells transfected with the shRNA-p53. **d** Expression of miR199a-3p mRNA in cardiac c-kit^+^ cells transfected with the shRNA-p53. *Con* control

It had been reported that P53 activates miR199a-3p expression at the post-transcriptional level [22]. We constructed a P53 lentivirus to infect cardiac c-kit^+^ cells; after transfection, P53 expression levels were examined by Western blotting (Fig. 8a). Indeed, overexpression of P53 increased miR199a-3p levels by approximately 3.8-fold (Fig. 8b).

We subsequently transfected cardiac c-kit^+^ cells with a shRNA-P53. Western blotting revealed that shRNA transfection decreased the P53 protein level 2.6-fold compared with that in shRNA-NC-treated cells (Fig. 8c). After transfection, knockdown of P53 decreased miR199a-3p expression (Fig. 8d).

These assays demonstrated that P53 induced miR199a-3p expression and, in turn, miR199-3p decreased P53 activity. Therefore, miR199a-3p and P53 are coupled through CABLES1 and comprise a negative feedback loop. Finally, the increase in miR199-3p may ultimately offset the P53 upregulation in heart failure.

## Discussion

The role of miR199a-3p as a tumor suppressor or oncogene has been studied by several groups [30–32]. miR-199a-3p has been found to be highly expressed in ovarian and breast cancer [33], but has low expression in hepatocellular carcinoma [34] and in bladder cancer [35]. Overexpression of miR199a-3p was found to inhibit the invasiveness of tumor cells [36] and to modulate their sensitivity to doxorubicin-induced apoptosis in hepatocellular carcinoma [37]. Furthermore, it was reported that miR199a-3p targeting of CABLES1 might play an important role in breast cancer tumor progression [38] and human osteosarcoma [31], as well as supporting its role as an oncomiR in a pre-leukemic mouse model [39]. Previous study showed that miR199a-3p has been found to be dysregulated in end-stage heart failure [40].

In cardiomyocytes, miR199a downregulates hypoxia-inducible factor 1 and the stabilization of the proapoptotic factor p53 [9], and is one of the factors regulating cell size; its overexpression in cardiomyocytes leads to hypertrophy [41]. Moreover, overexpression of miR199a in cardiomyocytes results in disruption of sarcomere structure and, conversely, suppression of miR199a abolishes this phenotype [15]. Overexpression of miR195 and -199a in cardiomyocytes also provoked myocyte enlargement [11]. Increased expression of miR199a-3p may be proapoptotic by virtue of its ability to downregulate the survival kinase ERK2 [36].

In our study, we have shown that miR199a-3p is significantly decreased in the plasma samples of heart failure patients compared with healthy donors. We found that expression of miR199a-3p promoted proliferation and survival of cardiac c-kit^+^ cells. To identify the potential targets of miR199a-3p that would facilitate cell growth, we used bioinformatic search tools. Computational screening showed that one of the potential target sites of miR199a-3p was in the 3’ UTR of mouse and human CABLES1.

CABLES1 is a novel cyclin-dependent kinase (CDK)-binding protein that maps to human chromosome 18q11-12 [26, 27]. In proliferating cells, CABLES1 connects Cdk2 and Wee1 which results in increased phosphorylation of Cdk2 at Y15, decreased kinase activity, and reduced cell proliferation. Loss of CABLES1 expression is observed with high frequency in human colon, lung, ovarian, and endometrial cancers [42–45], and also enhances tumor progression in the ApcMin/^+^ mouse model and activates the Wnt/β-catenin signaling pathway [46]. In addition to slowing cell proliferation, CABLES1 overexpression augments wild-type p53-induced cell death, suggesting Cables1 may contribute to apoptosis [28, 29].

This idea is also supported by the finding that mouse embryonic fibroblasts (MEFs) derived from CABLES1^–/–^ mice are more resistant to cell death induced by serum withdrawal when compared to MEFs derived from CABLES1^+/+^ mice [47]. Furthermore, CABLES1^–/–^ MEFs proliferate at a faster rate when compared to their CABLES1^+/+^ counterparts and exhibit delayed replicative senescence [47]. All the reported results show that CABLES1 serves as a negative regulator of cell proliferation, and that loss of CABLES1 function can lead to uncontrolled growth. In addition, rescue experiments further confirmed that miR199a-3p functioned through downregulating CABLES1 expression directly to promote cell proliferation and reduce cell apoptosis.

Suppression of p53 expression in CABLES1 morphants suggests that the phenotype of CABLES1 morphants is due in part to p53-dependent apoptosis [29]. Herein, we showed that the function of miR199a-3p to promote cell proliferation and inhibit cell apoptosis was also attributed to its repression of P53 protein expression. Meanwhile, the upregulation of p53 may be critical in the modulation of heart failure [20, 21]. To explore whether upregulated P53 expression was attributed to affected miR199a-3p expression in cardiac c-kit^+^ cells, we constructed P53 lentivirus to infect cardiac c-kit^+^ cells, and then detected that overexpression of P53 increased miR199a-3p levels. However, it has been reported that p53 upregulation leads to cardiac dilatation and heart failure [48]. We suppose that upregulation of p53 may the initial factor, and p53 elevated the miR199a-3p expression. The upregulation of miR199a-3p can promote cell proliferation and protects against angiotensin II-induced apoptosis. Interestingly, miR199a-3p was significantly decreased in the plasma of heart failure patients. The contradictory results indicate that there may be an opposite expression between cardiac c-kit^+^ cells and cardiomyocytes. The research on cardiomyocytes and miR199a-3p needs further investigation.

## Conclusion

**Fig. 9:**
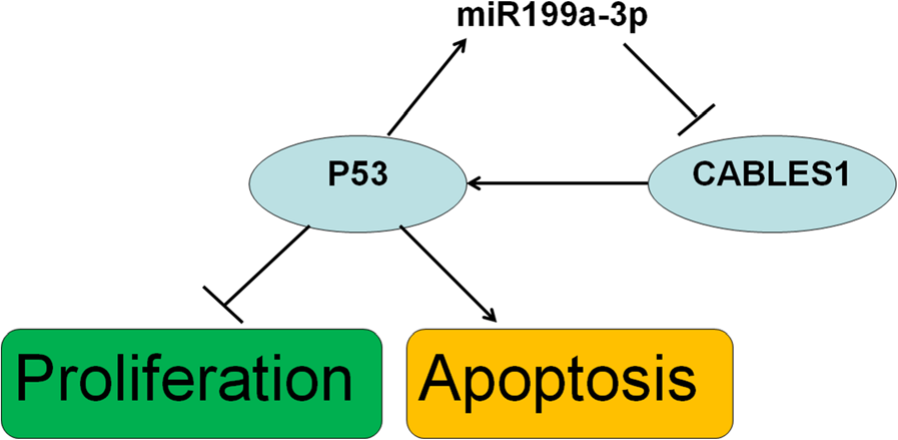
Schematic illustration of the feedforward/positive feedback loop between miR199a-3p, CABLES1, and P53. *CABLES1* Cdk5 and Abl enzyme substrate 1

Taken together, our data confirmed one new mechanism by which the downregulation of miR199a-3p miRNAs and the responsive upregulation of its target protein CABLES1 induced the P53 increase. The high expression of P53 ultimately offset the miR199a-3p upregulation, which provided evidence of a novel negative feedback loop that likely contributes to cardiac c-kit^+^ cell proliferation and apoptosis (Fig. 9).

## Abbreviations

AUC: Area under the curve
CABLES1: Cdk5 and Abl enzyme substrate 1
CDK: Cyclin-dependent kinase
DMEM: Dulbecco’s modified Eagle’s medium
EdU: 5-Ethynyl-2-deoxyuridine
FBS: Fetal bovine serum
HIF-1a: Hypoxia-inducible factor-1a
iPSC: Induced pluripotent stem cell
MEF: Mouse embryonic fibroblast
miRNA: MicroRNA
PBS: Phosphate-buffered saline
PI: Propidium iodide
qRT-PCR: Quantitative real-time polymerase chain reaction
RAAS: Renin–angiotensin–aldosterone system
ROC: Receiver-operating characteristic
SNS: Sympathetic nervous system
TU: Transducing units
UTR: Untranslated region
