# Correction to: miR199a-3p regulates P53 by targeting CABLES1 in mouse cardiac c-kit+ cells to promote proliferation and inhibit apoptosis through a negative feedback loop

**DOI:** 10.1186/s13287-019-1514-4

**Published:** 2019-12-20

**Authors:** Jingjin Liu, Yongshun Wang, Jinjin Cui, Meng Sun, Zhongyue Pu, Chao Wang, Wenjuan Du, Xinxin Liu, Jian Wu, Jingbo Hou, Shuo Zhang, Bo Yu

**Affiliations:** 1 Cardiology Department, Second Affiliated Hospital of Harbin Medical University, Harbin, 150086 Heilongjiang Province China; 2 Key Laboratories of the Education Ministry for Myocardial Ischemia Mechanisms and Treatment, Harbin, Heilongjiang Province China

**Correction to: Stem Cell Res Ther (2017) 8:127**

**https://doi.org/10.1186/s13287-017-0515-4**

After publication of our article [1] we became aware that there were errors in Fig. 5b and Fig. 6c, namely that the immunofluorescence of EDU-positive cells of the CABLES1 transfection group in Fig. 5b (panel 2) and the cell cycle distribution of the combination group (treatment with the antimiR199a-3p and shRNA-CABLES1) in Fig. 6c (panel 3) were incorrectly presented. These errors do not affect the discussion or conclusions in the article. The correct versions of Figs. 5 and 6 are shown below. We apologize to the journal and to readers for this error.

**Fig. 5:**
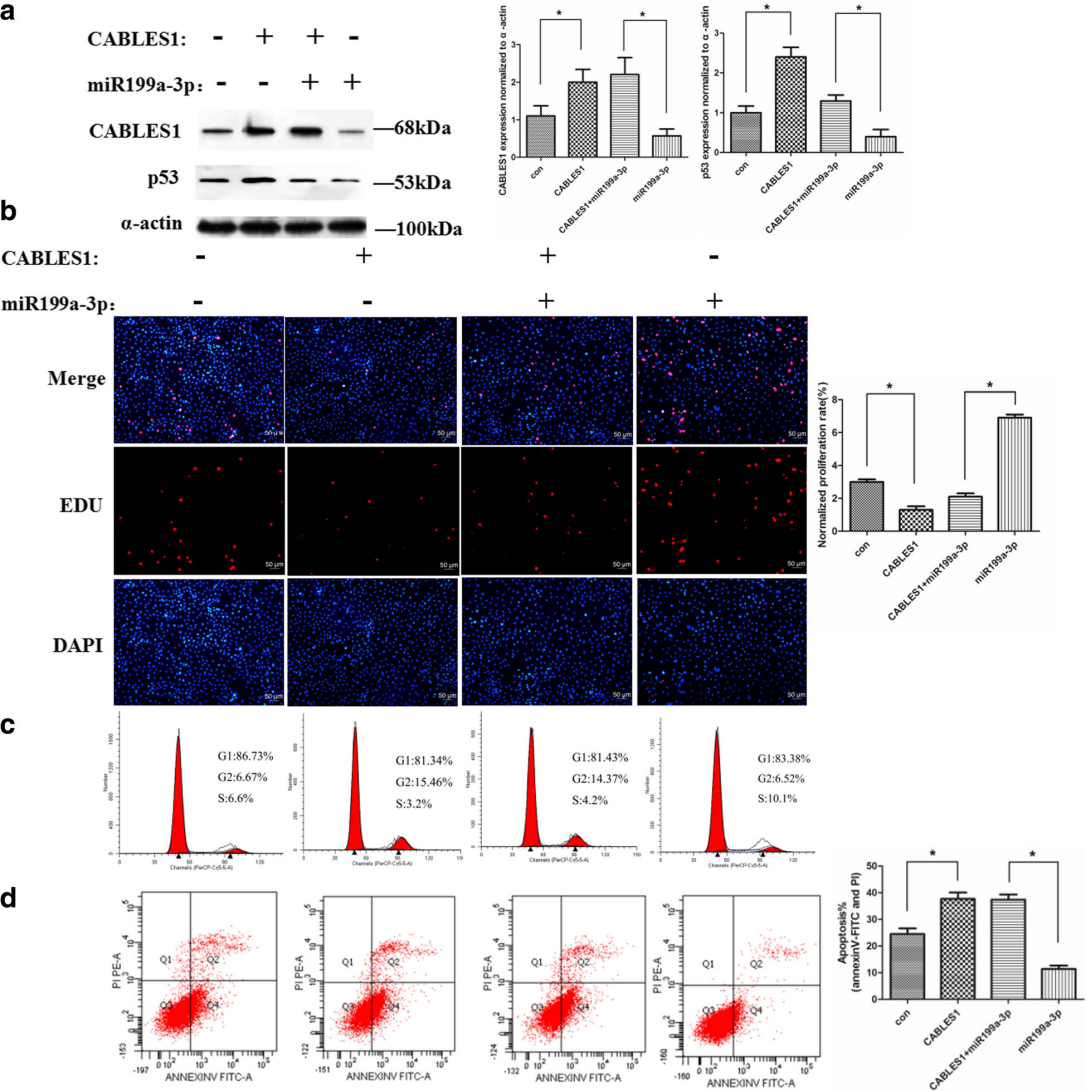
miR199a-3p regulates cell proliferation and apoptosis through targeting CABLES1. **a** Western blot analysis of proteins from cardiac c-kit+ cells transfected with the miR199a-3p and CABLES1 lentiviral vectors (*p < 0.05). The protein profiles were normalized to α-actin. **b** The cells were stained with EdU and DAPI (*p < 0.05). **c** Cell cycle distribution of cardiac c-kit+ cells after transfection with the miR199a-3p and CABLES1 lentiviral vectors. **d** Representative annexin V/PI flow cytometry analysis of cardiac c-kit+ cells and analysis of annexin V+/PI+ cardiac c-kit+ cells by flow cytometry. *CABLES1* Cdk5 and Abl enzyme substrate 1, *Con* control, *PI* propidium iodide (*p < 0.05)

**Fig. 6:**
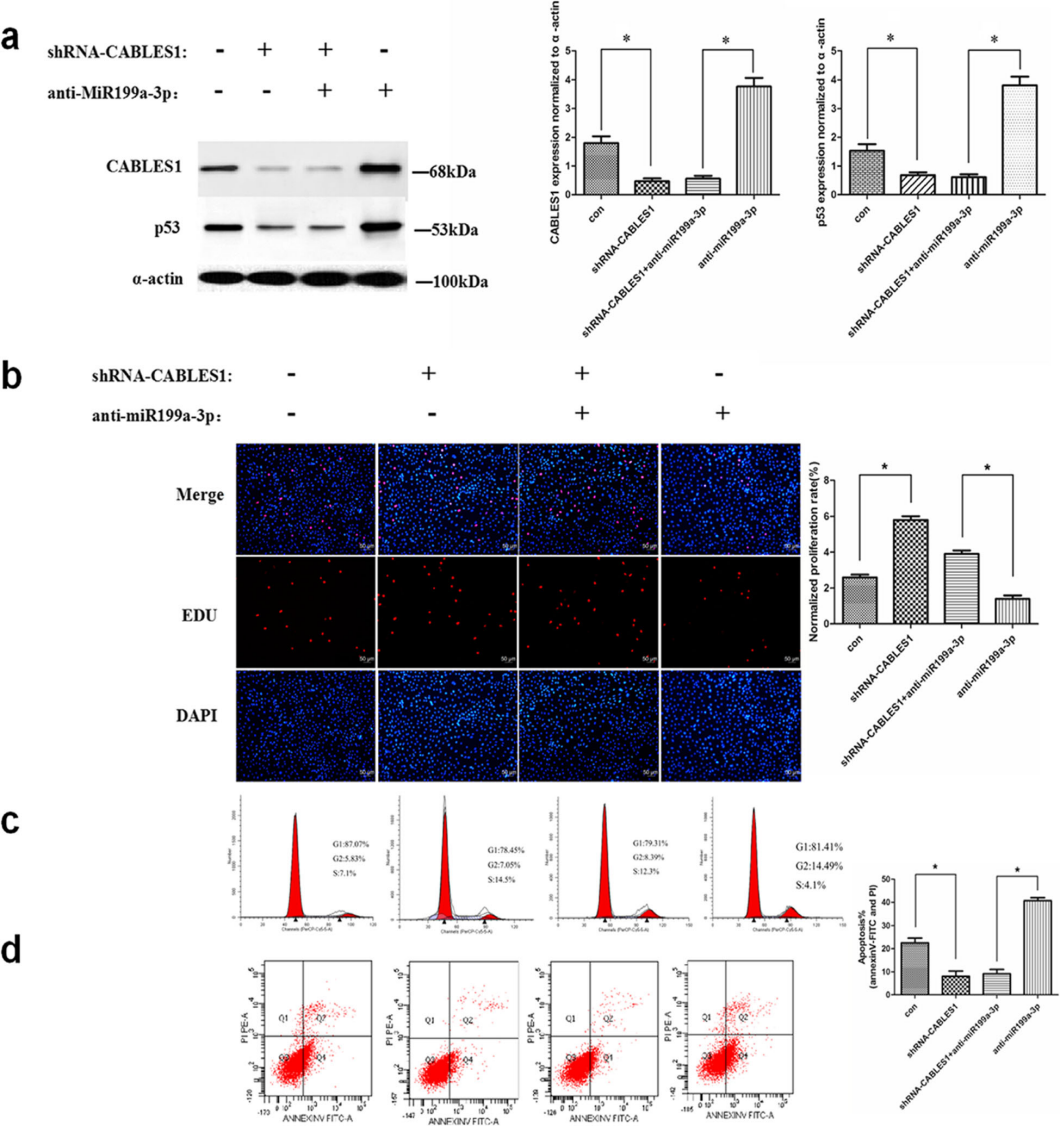
miR199a-3p regulates cell proliferation and apoptosis through targeting CABLES1. **a** Western blot analysis of proteins from cardiac c-kit+ cells transfected with the anti-miR199a-3p and shRNA-CABLES1 lentiviral vectors (*p < 0.05). **b** The cells were stained with EdU and DAPI (*p < 0.05). **c** Cell cycle distribution of cardiac c-kit+ cells after transfection with the anti-miR199a-3p and shRNA-CABLES1 lentiviral vectors. **d** Representative annexin V/PI flow cytometry analysis of cardiac c-kit+ cells and analysis of annexin V+/PI+ cardiac c-kit+ cells by flow cytometry (*p < 0.05). *CABLES1* Cdk5 and Abl enzyme substrate 1, *Con* control, *PI* propidium iodide

## References

[1] Liu J, Wang Y, Cui J, Sun M, Pu Z, Wang C, Du W, Liu X, Wu J, Hou J, Zhang S, Yu B (2017). miR199a-3p regulates P53 by targeting CABLES1 in mouse cardiac c-kit^+^ cells to promote proliferation and inhibit apoptosis through a negative feedback loop. Stem Cell Res Ther.

